# Targeting Serotonin Transporters in the Treatment of Juvenile and Adolescent Depression

**DOI:** 10.3389/fnins.2019.00156

**Published:** 2019-02-27

**Authors:** Melodi A. Bowman, Lynette C. Daws

**Affiliations:** ^1^Department of Cellular and Integrative Physiology, University of Texas Health Science Center at San Antonio, San Antonio, TX, United States; ^2^Department of Pharmacology, University of Texas Health Science Center at San Antonio, San Antonio, TX, United States

**Keywords:** serotonin, transporters, depression, antidepressants, development, children, juveniles, adolescents

## Abstract

Depression is a serious public health concern. Many patients are not effectively treated, but in children and adolescents this problem is compounded by limited pharmaceutical options. Currently, the Food and Drug Administration approves only two antidepressants for use in these young populations. Both are selective serotonin reuptake inhibitors (SSRIs). Compounding matters further, they are therapeutically less efficacious in children and adolescents than in adults. Here, we review clinical and preclinical literature describing the antidepressant efficacy of SSRIs in juveniles and adolescents. Since the high-affinity serotonin transporter (SERT) is the primary target of SSRIs, we then synthesize these reports with studies of SERT expression/function during juvenile and adolescent periods. Preclinical literature reveals some striking parallels with clinical studies, primary among them is that, like humans, juvenile and adolescent rodents show reduced antidepressant-like responses to SSRIs. These findings underscore the utility of preclinical assays designed to screen drugs for antidepressant efficacy across ages. There is general agreement that SERT expression/function is lower in juveniles and adolescents than in adults. It is well established that chronic SSRI treatment decreases SERT expression/function in adults, but strikingly, SERT expression/function in adolescents is increased following chronic treatment with SSRIs. Finally, we discuss a putative role for organic cation transporters and/or plasma membrane monoamine transporter in serotonergic homeostasis in juveniles and adolescents. Taken together, fundamental differences in SERT, and putatively in other transporters capable of serotonin clearance, may provide a mechanistic basis for the relative inefficiency of SSRIs to treat pediatric depression, relative to adults.

## Child and Adolescent Depression: Overview

Major depressive disorder, one of the most common forms of depression, affects individuals of all ages with approximately 5.7% of children and 11.3% of adolescents experiencing major depressive episodes (Fuhrmann et al., 2014; Mojtabai et al., 2016). Hallmarks of major depressive disorder include feelings of sadness for long periods, anhedonia, significant increases or decreases in weight, sleeping disturbances, psychomotor retardation or agitation, lethargy, feelings of helplessness and hopelessness, difficulty concentrating and/or decision making, and suicidal thoughts (American Psychiatric Association, 2013). Diagnosis of major depressive disorder is based on an individual having at least five of these symptoms for two or more weeks. However, symptoms of depression do not present themselves the same in all ages. Children and adolescents have a more pernicious onset of symptoms and experience more irritability than adults (Hazell, 2009).

Depression is a serious public health concern. Many patients are not effectively treated, but in children and adolescents this problem is compounded by limited pharmaceutical options (for review, see Bylund and Reed, 2007). Only two selective serotonin reuptake inhibitors (SSRIs), fluoxetine (Prozac), and escitalopram (Lexapro), are currently approved by the United States Food and Drug Administration (FDA) for use in these young populations. However, children and adolescents are less effectively treated by these antidepressants compared with adults, further exacerbating the situation (Tsapakis et al., 2008; Hetrick et al., 2010, 2012). Moreover, treatment response to escitalopram is especially poor in children and adolescents carrying a common variant of the serotonin transporter (SERT) gene, which confers reduced SERT functionality (Kronenberg et al., 2007). Given the high prevalence of adolescent depression, affecting 4–8% of the population with a potential lifetime incidence as high as 25% (Kessler et al., 2001; Cheung et al., 2005; Bujoreanu et al., 2011; Thapar et al., 2012; Maughan et al., 2013) and, of great concern, the high incidence of suicide in this young population (“the third leading cause of death in the 15–19 years age group”; Reed et al., 2008), there is a clear and urgent need to establish the neural mechanisms underlying these differences between children and adolescents on the one hand and adults on the other, with the goal to discover novel targets for the development of improved treatments.

In spite of numerous reports documenting that the antidepressant response of children and adolescents is less than that of adults (Hazell et al., 2002; Bylund and Reed, 2007; Tsapakis et al., 2008; Hetrick et al., 2010, 2012), studies investigating the underlying mechanisms are lacking. That the brain is still undergoing rapid development during childhood and adolescent periods (Lenroot and Giedd, 2006) no doubt contributes, at least in part, to different treatment responses between children and adolescents, and adults. However, the neural reasons for the age-dependency of antidepressant response remain poorly understood. SSRIs, the most commonly prescribed antidepressants, and the only class approved for children and adolescents, act by inhibiting serotonin uptake via SERT, the high-affinity transporter for serotonin. This focused review aims to synthesize current clinical and preclinical literature investigating the utility of the SSRI class of antidepressant in the treatment of childhood and adolescent depression, together with what is known about SERT expression and function during these developmental periods. Importantly, we discuss findings from preclinical studies that are beginning to shed light on putative mechanisms accounting for the relatively poor response of children and adolescents to SSRIs relative to adults.

## Available Treatments

The safety and effectiveness of many antidepressants for use in children and adolescents have been extensively studied and reviewed (e.g., Maneeton and Srisurapanont, 2000; Hazell et al., 2002; Courtney, 2004; Hazell, 2004; Whittington et al., 2004; Bhatia and Bhatia, 2007). Fluoxetine is FDA approved for children and adolescents as young as 8 years and older, and escitalopram is approved for children and adolescents age 12 and older. In addition to these, other antidepressants can be prescribed off label. For example, sertraline and fluvoxamine (also SSRIs) are approved to treat obsessive compulsive disorder in children and adolescents and therefore can be prescribed “off label” to treat depression in these young populations (Kodish et al., 2011). The numbers of prescriptions for antidepressants continues to increase in individuals over the age of six with approximately 3% of individuals under 18 years of age being prescribed an antidepressant (Kafali et al., 2018).

Whether these treatments yield clinical benefits remains controversial (see Table 1). For example, fluoxetine was originally found to be effective in treating children and adolescents with major depressive disorder (Emslie et al., 1997, 2002), but using fluoxetine as a positive control in later studies examining the efficacy of other SSRIs, the same investigators found no difference between fluoxetine and placebo treated groups (Atkinson et al., 2014; Emslie et al., 2014). As summarized in Table 1, randomized controlled trials conducted in children and adolescents have yielded variable results across studies and types of SSRIs, with roughly equal numbers reporting therapeutic benefit versus no difference from placebo. These mixed results are likely attributable, at least in part, to differences in population samples and symptomology scales used, with no clear pattern emerging with regard to dose or duration of treatment. In turn, this highlights a need to refine and standardize the diagnostic criterion for depression in juveniles and adolescents, which likely differs from that used to assess depression in adults. Regardless, based on current clinical literature it is difficult to conclude whether or not SSRIs are beneficial in the treatment of depressive disorders in children and adolescents. To this end, preclinical studies are beginning to provide insights into the utility of SSRIs as antidepressants in these young populations.

**Table 1:** Clinical studies examining the efficacy of antidepressant medications in children and adolescents.

Age (years)	SSRI	Dose and duration	Outcome	Reference
13–18	Citalopram	10 mg/day12 weeks	No difference from placebo	von Knorring et al., 2006
7–17	Citalopram	20–40 mg/day8 weeks	Improved treatment compared to placebo	Wagner et al., 2004
7–17	Duloxetine	60–120 mg/day36 weeks	No difference from placebo	Atkinson et al., 2014
7–17	Duloxetine	30, 60, 90, or 120 mg/day36 weeks	No difference from placebo	Emslie et al., 2014
12–17	Escitalopram	10 or 20 mg/day8 weeks	Improved treatment compared to placebo	Emslie et al., 2009
12–17	Escitalopram	10–20 mg/day24 weeks	Improved treatment compared to placebo	Findling et al., 2013
6–17	Escitalopram	10–20 mg/day8 weeks	No difference from placebo	Wagner et al., 2006
7–17	Fluoxetine	20 mg/day8 weeks	Improved treatment compared to placebo	Emslie et al., 1997
8–18	Fluoxetine	10 or 20 mg/day9 weeks	Improved treatment compared to placebo	Emslie et al., 2002
12–17	Fluoxetine	10–40 mg/day12 weeks	Improved treatment compared to placebo	March et al., 2004
13–18	Fluoxetine	20, 40, or 60 mg/day7 weeks	No difference from placebo	Simeon et al., 1990
13–18	Paroxetine	20–40 mg/day12 weeks	No difference from placebo	Berard et al., 2006
7–17	Paroxetine	10–50 mg/day8 weeks	No difference from placebo	Emslie et al., 2006
12–18	Paroxetine	20–40 mg/day8 weeks	Improved treatment compared to placebo	Keller et al., 2001
6–17	Sertraline	50–200 mg/day6 weeks	Improved treatment compared to placebo	Alderman et al., 1998
6–17	Sertraline	50–200 mg/day10 weeks	Improved treatment compared to placebo	Wagner et al., 2003
12–17	Vilazodone	15 or 30 mg/day8 weeks	No difference from placebo	Durgam et al., 2018

## Lessons Learned From Preclinical Studies

Preclinical studies using murine models afford well-controlled conditions to study normal brain development, as well as the neural and behavioral effects of SSRIs, and other classes of antidepressants, in juveniles and adolescents. While prior to 2008 only a handful of studies had examined antidepressant-like activity of SSRIs in adolescent rodents (and none in juveniles), research in this arena has been steadily increasing during the past decade (see Table 2). These studies are discussed in the following sections.

**Table 2:** Preclinical studies examining the antidepressant-like effects of SSRIs (2A), effect of chronic SSRI treatment on SERT expression (2B), and age-dependent shifts in SERT expression (2C) in juvenile and adolescent rodents, compared to adults.

A: Behavioral outcomes from studies of antidepressant-like activity								

Age	Species	Strain	Sex	Drug	Dose/duration	Assay	Outcome	Reference
**Behavioral studies – acute administration**								
P21 compared with adult (age not specified)	Rat	Sprague-Dawley	Male	Escitalopram (ESC) Desipramine (DMI) Fluoxetine (FLX) Imipramine (IMI)	1–20 mg/kg 1–20 mg/kg 0.1–10 mg/kg 0.1–10 mg/kg Drugs given ip 23 h, 5 h, and 1 h prior to FST	FST	FLX and ESC produced similar, dose-dependent antidepressant-like effects in P21 and adults rats, however, DMI and IMI were without effect in both ages. Both ESC and DMI produced antidepressant-like effects in adult rats (FLX and IMI were not tested in adults).	Reed et al., 2008
P21 and P28 compared with P90	Mouse	C57BL/6J	Male and Female	Escitalopram (ESC) Desipramine (DMI)	10 mg/kg, sc 32 mg/kg, ip Drugs given 30 min prior to test	TST	Antidepressant-like effects were observed in all ages. However, ESC was less effective in P21 mice compared to P28 and P90 mice. No sex differences.	Mitchell et al., 2013
P21 and P28 compared with P90	Mouse	C57BL/6J SERT+/+ SERT+/- SERT-/-	Male and Female	Escitalopram (ESC)	0.32–10 mg/kg, sc, given 30 min prior to test	TST	Antidepressant-like effects were observed in all ages. Maximal effects were less in P21 mice than in P90 mice, and more so in SERT+/- mice. The potency for ESC to produce antidepressant-like effects in SERT+/+ and SERT+/- mice was greater in P21 and P28 mice than in adults. No effect of ESC in SERT-/- mice. No sex differences.	Mitchell et al., 2016
P28 compared with P84, P168, and P280	Mouse	Swiss	Male	Citalopram (CIT) Paroxetine (PRX) Imipramine (IMI) Desipramine (DMI) Bupropion (BUP) Moclobemide (MOC)	2–32 mg/kg 1–8 mg/kg 4–32 mg/kg 2–16 mg/kg 4–64 mg/kg 8–128 m/kg Drugs given ip 30 min prior to test	FST	At low doses, CIT and PRX reduced immobility time in P280 mice, but not younger mice. At higher doses, CIT and PRX were inactive in P280 mice, but active in younger mice. IMI, DMI and BUP reduced immobility in all age groups. MOC reduced immobility only at the highest dose, and only in P84 mice.	Bourin et al., 1998
P28 compared with P280	Mouse	Swiss	Male	Fluvoxamine (FLV) Sertraline (SER) Venlafaxine (VEN) Imipramine (IMI) Maprotiline (MAP)	4–16 mg/kg 8–32 mg/kg 4–16 mg/kg 8–32 mg/kg 8–32 mg/kg Drugs given ip 30 min prior to test	FST	At higher doses, all antidepressants were effective in both ages, with the exception of FLV in P280 mice. Reduction in time spent immobile was greater in P28 mice than P280 mice following SER, IMI and MAP.	David et al., 2001
P35 compared with P84–91	Mouse	C57BL/6J F2 BALB/cJ	Male	Fluoxetine (FLX) Imipramine (IMI)	10 and 20 mg/kg 10 and 30 mg/kg Drugs given ip 30 min prior to TST, and 24 h, 18 h, and 1 h prior to FST.	TST and FST	FLX produced an antidepressant-like effect in F2 and C57 P35 and adult mice but not BALB/cJ P35 and adult mice in the TST. In the FST, FLX produced an antidepressant-like effect in adult mice of all strains, however, only in F2 juvenile mice. IMI produced an antidepressant-like effect across all strains and ages of mice in both tests.	Mason et al., 2009
**Behavioral studies – chronic administration**								
P21 and P28 compared with adult (age not specified)	Rat	Sprague-Dawley	Male	Escitalopram (ESC) Desipramine (DMI)	10 mg/kg 3–15 mg/kg All drugs administered ip twice per day for 7 days	Inescapable shock and shuttle box test	ESC produced an antidepressant-like effect in all ages. DMI did not produce an effect in P21 rats, but did in P28 and adult rats.	Reed et al., 2009
P25 compared with P65	Rat	Wistar	Male	Fluoxetine (FLX)	5 mg/kg, ip for 3 weeks with a 1 week washout	FST	No antidepressant-like effect in either age.	Bouet et al., 2012
P25–49 compared with P67–88 at start of treatment	Rat	Wistar	Male	Fluoxetine (FLX)	12 mg/kg, oral gavage for 21 days	FST, 10 days after last dose.	FLX increased immobility time in adolescents, and had no effect in adults.	Homberg et al., 2011
P28–49 compared with P70–91	Rat	Wistar	Male	Paroxetine (PRX)	5 and 10 mg/kg, drinking water for 18 days	FST	PRX did not produce an antidepressant-like effect in adolescent rats, however, it did in adult rats.	Karanges et al., 2011

**B: SERT expression after chronic SSRI treatment**								

**Age**	**Species**	**Strain**	**Sex**	**Drug**	**Dose and Duration**	**Assay**	**Outcome**	**Reference**

P25 compared with P50	Rat	Wistar	Male	Fluoxetine (FLX)	5 mg/kg, drinking water for 2 weeks	[^3^H]paroxetine binding assay	Increase in SERT expression in the frontal cortex in rats treated with FLX starting at P25, however, not in rats treated with FLX starting at P50.	Wegerer et al., 1999
P25 compared with P65	Rat	Wistar	Male	Fluoxetine (FLX)	5 mg/kg, ip for 3 weeks with a 1 week washout	*Ex vivo* binding assay with [^123^I]β-citalopram and *in vivo* pharmacological MRI (phMRI)	In P25 rats, there was an increase in binding after FLX treatment in the prefrontal cortex and hippocampus. In P65 rats, there was a decrease in binding in the occipital and cingulate cortex after treatment with FLX. phMRI did not indicate changes in level of activation of brain areas after treatment with FLX in either P25 or P65 rats.	Bouet et al., 2012
P28–49 compared with P70–91	Rat	Wistar	Male	Paroxetine (PRX)	5 and 10 mg/kg, drinking water for 18 days	Autoradiography with [^125^I]RTI-55	SERT density in the basolateral amygdala was increased in adolescent rats treated with PRX compared to control, but not in adults. There were no differences in SERT density in the CA3 of the hippocampus between rats treated with PRX and control in adolescent and adult rats.	Karanges et al., 2011
Two-year old^∗^	Monkey	Rhesus	Male	Fluoxetine (FLX)	3 mg/kg/day for 1 year in mashed banana, with a 1.5 year washout	Positron emission tomography (PET) with [^11^C]DASB	SERT expression was increased in neocortex, hippocampus, lateral temporal and cingulate cortices.	Shrestha et al., 2014

**C: Post-natal ontogeny of SERT**								

**Age**	**Species**	**Strain**	**Sex**			**Assay**	**Outcome**	**Reference**

P0, P7, P14, P21, P28, P70	Rat	Wistar	Male			Quantitative autoradiography with [^3^H]*N,N*-dimethyl-2-(2-amino-4-methylphenylthio)benzylamine (MADAM)	In terminal regions such as amygdala and hypothalamus, expression peaked around P21, decreased at P28 and plateaued through P70.	Galineau et al., 2004
P21 and P28 compared with P90	Mouse	C57BL/6J	Male and female			[^3^H]Citalopram saturation binding in hippocampal homogenates	There was no difference in [^3^H]citalopram maximal binding or affinity across ages. However, there was significantly greater variability in affinity for [^3^H]citalopram binding in P21 mice compared to P28 and P90 mice.	Mitchell et al., 2013
P21 and P28 compared with P90	Mouse	C57BL/6J	Male and female			Quantitative autoradiography with [^3^H]citalopram	SERT expression increased with age in terminal regions and decreased with age in cell body regions.	Mitchell et al., 2016
P24–32 and P40–41 compared with 3–5 months and 12–14 months	Rat	Wistar	Male			Immunofluorescence	Prepubertal (P24–32) and pubertal (P40–41) rats exhibited lower SERT immunoreactivity in the lateral septum and dorsal raphe compared to young adult (3–5 months) rats with no difference in SERT immunoreactivity compared to middle age rats.	Ulloa et al., 2014
P25 compared with P65	Rat	Wistar	Male			*Ex vivo* binding assay with [^123^I]β-citalopram and phMRI	[^123^I]β-citalopram binding in the prefrontal cortex and cingulate cortex was lower in P25 rats compared to P65 rats, however, it was higher in the raphe nuclei of P25 rats compared to P65 rats.	Bouet et al., 2012
P28–49 compared with P70–91	Rat	Wistar	Male			Autoradiography with [^125^I]RTI-55	There were no significant differences between adolescents and adults in [^125^I]RTI-55 binding in either BLA or CA3 region of hippocampus.	Karanges et al., 2011

*^∗^Note that puberty occurs in Rhesus macaques between 2.5 and 4.5 years (Colman et al., 2009).*

### Antidepressant-Like Efficacy of SSRIs in Juvenile and Adolescent Rodents

In rodents, postnatal days (P) 21–27 are considered the juvenile period, P28–42 early adolescence and P44–56 late adolescence (Spear, 2000; Bylund and Reed, 2007; Hefner and Holmes, 2007; Pechnick et al., 2008; Reed et al., 2008). Although there are still relatively few publications investigating antidepressant-like efficacy of SSRIs in juvenile and adolescent rodents, the literature is growing, and revealing some interesting patterns, discussed below (Table 2).

#### Acute Antidepressant-Like Effects of SSRIs

Studies examining the acute effects of antidepressant drugs have used the forced swim test (FST) and/or tail suspension test (TST), the gold standards for assaying antidepressant-like activity in rodents (Cryan and Holmes, 2005; Castagné et al., 2011). While there are some inconsistencies among reports (Table 2A), the most common finding is that SSRIs are less effective in juvenile and adolescent rodents, than in adults (Bourin et al., 1998; Mason et al., 2009; Mitchell et al., 2013, 2017). This is in line with clinical reports (Tsapakis et al., 2008; Hetrick et al., 2010, 2012), and underscores the utility of these preclinical tests as predictors of antidepressant-like efficacy in humans.

Interestingly, Mason et al. (2009) found that age-dependent differences in antidepressant-like response were also dependent on mouse strain and test used. For example, in the TST fluoxetine (20 mg/kg) produced an antidepressant-like effect in juvenile (P35) and adult C57BL/6J and F2 mice, but not in BALB/C mice. However, when using the FST, juvenile and adult C57BL/6 mice were insensitive to fluoxetine, but F2 and BALB/C mice responded. This is not surprising in view of elegant studies from Lucki’s group using adult mice to show clear strain-dependent differences in response to fluoxetine in the FST (Lucki et al., 2001) and to citalopram in the TST (Crowley et al., 2005). Regardless, in the Mason study, when antidepressant effects were detectable they were less in juveniles than in adults when using the FST, and less than or equivalent to those of adults when using the TST.

While the majority of studies have used mice, one study used rats (Reed et al., 2008). Using the FST, these investigators found that the SSRIs fluoxetine and escitalopram produced antidepressant-like effects that were of a similar magnitude in P21 and adult rats (Table 2A). Interestingly, they found that the tricyclic antidepressants, desipramine and imipramine, were ineffective in P21 rats, but produced robust antidepressant-like effects in adults. While the mechanistic underpinnings for these results are yet to be determined, a common view is that delayed maturation of the norepinephrine neurotransmitter system relative to serotonin is a factor (Bylund and Reed, 2007). However, in contrast to the rat study, mouse studies generally found tricyclic antidepressants to be either equally effective in producing antidepressant-like effects in the TST and FST in juveniles, adolescents, and adults (Bourin et al., 1998; Mason et al., 2009; Mitchell et al., 2013), or more efficacious in juveniles and adolescents than in adults (David et al., 2001; Mitchell et al., 2017), suggesting that in mice, the norepinephrine neurotransmitter system matures faster than in the rat. In humans, tricyclics are not prescribed for children and adolescents due to poor tolerability (e.g., vertigo, tremors, low blood pressure, and dry mouth; Andersen et al., 2009; Dell’Osso et al., 2011; Hazell and Mirzaie, 2013). However, preclinical findings from mouse studies indicate that the utility of NET, and/or dual NET and SERT blockers in the treatment of juvenile and adolescent depression warrants further investigation. It is possible that the development of new NET (and NET/SERT) targeting drugs that have fewer side effects than currently available drugs may be better suited to the treatment of pediatric depression (Mitchell et al., 2017).

Clearly, differing rates of development of noradrenergic and serotonin systems, and the development of systems that impinge on them (e.g., histamine, see Munari et al., 2015) may also factor into the magnitude of antidepressant-response as a function of age. Rates of maturation also differ among species, and is a consideration when relating findings from rodents to outcomes in humans. The reader is directed to an excellent review by Murrin et al. (2007) for information on differential rates of development of noradrenergic and serotonergic systems among species. While studies in human are relatively scant, those that exist, together with studies in non-human primates, suggest that the serotonin system matures faster than the noradrenergic system (Murrin et al., 2007). It will be of interest for future studies to precisely map treatment response to different classes of antidepressant on developmental stage of the noradrenergic and serotonergic systems in humans.

Lastly, one study included constitutive SERT heterozygote mice as a murine model of humans carrying a common variant of the SERT gene, which confers reduced SERT functionality, and who respond less well to SSRIs than those without this variant (Kronenberg et al., 2007). Consistent with clinical literature, the maximal effect of escitalopram in juvenile SERT heterozygote mice was less than that in juvenile SERT wildtype mice. Moreover, in both SERT genotypes the maximal effect in juveniles was less than that in adults (Mitchell et al., 2016). These findings again underscore the utility of preclinical models of antidepressant-like activity in probing mechanisms contributing to age- and genotype-dependent responses to antidepressants (but see section “Caveats to Currently Available Behavioral Assays for Identifying Drugs With Antidepressant Potential”).

In sum, the most common finding from studies of the acute antidepressant-like effects of SSRIs in preclinical studies is that they are less effective in juveniles and adolescents than in adults, though some studies do report no difference in response among ages. With proper attention to mouse strain, and behavioral test employed, such preclinical models provide valuable tools to study the mechanistic basis for SSRIs being less effective in treating pediatric depression than in adults, as will be discussed further.

#### Chronic Antidepressant-Like Effects

While the TST and FST are currently the best tests available to quickly screen for drugs with antidepressant potential, for drugs to be useful clinically they must be effective after chronic administration. Numerous groups have reported antidepressant-like effects following chronic SSRI treatment in adult mice and rats, using tests such as the FST, TST, and novelty-induced hypophagia (Detke et al., 1997; Dulawa et al., 2004; Cryan et al., 2005; Holick et al., 2008; Miller et al., 2008; Balu et al., 2009; Jiao et al., 2011; Roohi-Azizi et al., 2018), with only one group reporting no effect (Griebel et al., 1999). However, only four studies have examined the antidepressant-like effect of chronic SSRI administration in juveniles and/or adolescents. All have used rats (Table 2A). Three used the FST, and though different SSRIs were used (fluoxetine in two studies [Homberg et al., 2011; Bouet et al., 2012] and paroxetine in the other [Karanges et al., 2011]), the overall findings were in general agreement. In the two studies where there was no washout period, chronic SSRI treatment either had no effect in young rats (Karanges et al., 2011), or increased immobility time (i.e., pro-depressive-like behavior; Homberg et al., 2011), whereas in adults there was either no effect (Homberg et al., 2011), or a robust antidepressant-like effect (Karanges et al., 2011), in agreement with published literature using adult rodents. These results are consistent with clinical literature reporting that children and adolescents respond poorly to SSRIs compared with adults, if at all (Tsapakis et al., 2008; Hetrick et al., 2010, 2012). In the one study where a 1-week washout period was allowed before testing in the FST, no antidepressant-like effect of fluoxetine was observed in either juvenile/adolescent or adult rats (Bouet et al., 2012).

Another study utilized inescapable shock and shuttle box tests to assay antidepressant-like activity following a week of twice daily injections of escitalopram or desipramine in rats. Escitalopram produced similar antidepressant-like effects in juveniles, adolescents, and adults, but desipramine was only effective in adolescent and adult rats, and ineffective in juvenile rats (Reed et al., 2009). This result is consistent with findings reported by the same group following acute administration of these drugs (Reed et al., 2008; see discussion above).

Overall, results from studies examining the chronic effects of SSRIs in rats again support the utility of preclinical assays such as these. However, given that mice seem to more reliably parallel human clinical literature, at least in the acute tests described above, it will be of interest to implement mice in studies of chronic SSRI administration as well.

#### Caveats to Currently Available Behavioral Assays for Identifying Drugs With Antidepressant Potential

Though the FST and TST are the current gold standards for assaying antidepressant-like activity in rodents (Cryan and Holmes, 2005), it is important to note that while the vast majority of antidepressant drugs reduce immobility time in these tests, not all drugs that reduce immobility time are antidepressants. Thus, these tests are valuable when comparing the sensitivity to drugs across ages, but they do not necessarily predict therapeutic effectiveness in the treatment of depression. In this realm, the field is in need of new tests (be they behavioral or biochemical) that can reliably predict therapeutic efficacy of novel drugs for the treatment of depression, while screening out false positives.

In addition, to date studies of juvenile and adolescent rodents in tests for antidepressant-like activity of drugs have used only naïve animals. Ideally, such tests should be performed in animal models of depression, such as those afforded by unpredictable chronic mild stress, or social defeat paradigms, to name a few (see O’Leary and Cryan, 2013 for review). While these models have been frequently applied to studies of depression in adult rodents, their application to juveniles (in particular), as well as adolescents, is impractical due to the shortness of these developmental periods (e.g., 7-day juvenile period, 14-day early adolescent, and 14-day late adolescent periods; Spear, 2000; Bylund and Reed, 2007; Hefner and Holmes, 2007; Pechnick et al., 2008; Reed et al., 2008). This is further compounded when the effects of chronic drug treatment need to be studied. While it is not within the scope of this focused review to discuss the pros and cons of existing models for depression research, the reader should be aware that there are limitations to those currently available (see O’Leary and Cryan, 2013 for review). Of course, recapitulating the heterogeneous nature of human depression, without yet fully understanding the etiologies of this complex disorder, makes developing improved animal models a challenging undertaking. However, as we learn more about the complexities of this disorder, new knowledge can be applied to the development of improved models to study depression and to screen novel compounds for therapeutic efficacy in its treatment. Importantly, models that can be applied to the study of juveniles and adolescents are sorely needed.

### Expression and Function of SERT in Juvenile and Adolescent Rodents

Understanding how SERT expression and function varies across juvenile, adolescent and adult ages, and how chronic treatment with SSRIs influences SERT expression and function across these ages may lend insight into age-dependent differences in antidepressant-like responses to SSRIs. Studies investigating this subject are discussed below.

#### Effect of Chronic SSRI Treatment on SERT Expression and Function

In adult rodents, chronic treatment with SSRIs downregulates SERT expression (Benmansour et al., 1999; Gould et al., 2003, 2007; Mirza et al., 2007; Lin et al., 2017), which is associated with putatively reduced functionality of SERT, as demonstrated by *in vivo* chronoamperometry studies measuring clearance of serotonin from extracellular fluid in hippocampus (Benmansour et al., 1999). These decreases were not associated with reduced SERT gene expression or neurotoxicity (Benmansour et al., 1999). Several lines of evidence suggest that SSRI-induced downregulation of SERT function is attributable, at least in part, to internalization of SERT to the cytosolic compartment. For example, *in vitro* studies using Caco-2 cells transfected with human (h) SERT show that long-term exposure to fluoxetine causes internalization of hSERT, leaving less hSERT on the plasma membrane (Iceta et al., 2007). These studies showed no effect of fluoxetine treatment on either total hSERT protein or mRNA. Studies in rats found that chronic, but not acute fluoxetine treatment causes internalization of SERT in both cell bodies and terminals (Descarries and Riad, 2012). Similarly, translational approaches using stem cell-derived serotonergic neurons and a transgenic mouse expressing hSERT found that citalopram dose-dependently causes internalization of hSERT in both models (Matthäus et al., 2016). Such studies underscore the utility of complementary/translational approaches to understanding antidepressant response on a cellular and molecular level. While it remains to be determined if internalization of SERT following chronic SSRI treatment occurs in humans, and is temporally synced with therapeutic benefit, studies in adult rodents, and *in vitro*, suggests that downregulation of SERT, putatively by trafficking away from the plasma membrane, is at least in part required for ultimate therapeutic benefit following chronic treatment with SSRIs. Moreover, that it takes chronic exposure to SSRIs for this downregulation of SERT to occur, provides a potential mechanistic basis for the delay to onset of therapeutic benefit.

Currently there is no literature reporting effects of chronic SSRI treatment in juvenile rodents. This is almost certainly due to the very narrow juvenile window in rodents (i.e., P21–27). There is remarkably sparse literature on the effect of chronic SSRI treatment on SERT expression in adolescent rodents (reviewed in Daws and Gould, 2011). However, a consistent finding is that chronic treatment with SSRIs, beginning at either P25 or P28 in rodents, *increases* SERT expression in a number of brain regions (Wegerer et al., 1999; Karanges et al., 2011; Bouet et al., 2012) (Table 2B). These findings in rodents are further supported by a study in juvenile rhesus macaque monkeys, which found that chronic treatment with fluoxetine increased SERT expression in several brain regions, including neocortex and hippocampus (Shrestha et al., 2014) (Table 2B). With more SERT putatively becoming available to take up serotonin as SSRI treatment continues, the increases in extracellular serotonin that are thought to be needed to trigger the downstream cascade of events leading to ultimate therapeutic benefit could be greatly diminished. Thus, the clinical implications of increased SERT expression in children and adolescents could include a need to increase dose of SSRI in order to fully occupy SERT. It is interesting to note that initiating dosing of SSRIs for pediatric depression is generally less than that for adults. For example, initiating low doses of fluoxetine and citalopram to 5- to 12-year olds is less than 10 mg/d, whereas in adults (18–64 years) the dose is less than 20 mg/d (Bushnell et al., 2016). Before the black-box warning on all antidepressants was issued by the FDA in 2004, the percent of patients initiating low dose SSRI treatment was similar across ages (∼15%), with the exception of 10- to 12-year olds where the percent was much lower (5%). In contrast, following the black-box warning the percent of patients initiating low dose SSRI *(relevant to their age)* increased markedly (5–9 years to ∼28%, 13–17 years to ∼38%, 18–24 years to ∼25%, 25–64 years to ∼20%, and 10–12 years to ∼11%; see Bushnell et al., 2016). In the context of the present review, this raises the possibility that increasing initial dosing in pediatric depression may be therapeutically beneficial, though clearly with this comes the risk of increased, potentially life-threatening, side-effects. It must also be kept in mind that of the clinical studies summarized in Table 1, SSRI doses much higher than these initiating low doses (e.g., 60 mg/day for fluoxetine; 200 mg/d sertraline) yielded mixed outcomes, some reporting therapeutic efficacy, and others no difference from placebo.

Clearly it is difficult to draw conclusions from existing literature. The important take home message here is that *increased* SERT expression following chronic SSRI treatment in children and adolescents could account, at least in part, for their therapeutic efficacy being less than that in adults. Though there are no studies of SERT in brain of children and adolescents, one study examining platelet SERT expression before and after treatment with the SSRI sertraline, provides support for this premise. Sertraline treatment was found to decrease [^3^H]paroxetine binding affinity. Notably *increased* maximal binding of [^3^H]paroxetine to SERT was associated with non-response (Sallee et al., 1998). Further study into the benefits of more rapidly ramping SSRI dosing in pediatric depression may therefore be warranted.

To stay within the scope of this focused review, we have discussed only the consequences of chronic SSRI treatment for their primary target, SERT. Of course this is an overly simplistic view of the neural consequences of chronic SSRI treatment in juveniles and adolescents, which has also been shown to impact glycogen synthase kinase-3 (GSK3; Beurel et al., 2012), extracellular signal-regulated protein kinase 1/2 (ERK) and cAMP response element binding protein (CREB; Alcantara et al., 2014), brain derived neurotrophic factor (BDNF) and TrkB receptors (Kozisek et al., 2008), genes associated with cell cycle and survival (Tsapakis et al., 2014), and neuroplasticity (Bastos et al., 1999; Norrholm and Ouimet, 2000; Bock et al., 2013; Klomp et al., 2014). Some of the effects of chronic SSRI treatment on these factors in juveniles and adolescents are in parallel with those reported in adults (Bastos et al., 1999), while others are not (Kozisek et al., 2008; Beurel et al., 2012; Bock et al., 2013; Alcantara et al., 2014; Klomp et al., 2014). A deeper focus on SSRI-induced changes that are specific to juveniles and adolescents will help guide future research to develop improved antidepressants for these young populations.

#### Expression of SERT in Juveniles, Adolescents, and Adults

Prior to 2011, remarkably little was known about the ontogeny of SERT expression during juvenile and adolescent periods (reviewed in Daws and Gould, 2011). The first paper to describe postnatal ontogeny of SERT found that in terminal regions, including amygdala and hypothalamus, expression was maximal around P21, decreased at P28, before rising again to stabilize through P70, the oldest age tested (Galineau et al., 2004; Daws and Gould, 2011). Subsequent studies of SERT expression from P21 through adulthood reported either no change across those ages (Karanges et al., 2011; Mitchell et al., 2013), or a steady increase from P21 through adulthood in terminal fields (Bouet et al., 2012; Ulloa et al., 2014; Mitchell et al., 2016), and a decrease from P21 in cell body regions (Bouet et al., 2012; Mitchell et al., 2016) (Table 2C). Of particular interest, Mitchell et al. (2013) using [^3^H]citalopram saturation binding assays in hippocampal homogenates, provided evidence that SERT in juveniles may be functionally less able to transport serotonin than in adults. The modest variation in results among these studies is likely due to nuanced differences in the assays used, ages studies, and species used. However, taken together current evidence suggests that SERT expression and/or function in terminal fields is lower in juveniles than in adults, whereas expression in cell bodies is greater (Table 2C).

It is difficult to speculate on how these postnatal shifts in SERT expression and function ultimately play into therapeutic responsiveness to SSRIs, but this is clearly a line of investigation that warrants further research. Likewise, it will be important to determine the relationship between receptor systems that are well-known to be influenced by SSRIs (e.g., Artigas, 2013; Kulikov et al., 2018), SERT, and therapeutic response in juveniles and adolescents.

### Alternative Transporter Targets for Therapeutic Intervention in Juveniles and Adolescents

In recent times, there has been increasing recognition of transporter “promiscuity” (Daws, 2009). High-affinity monoamine transporters are not loyal to their specific monoamine, and will take up other species, sometimes with greater efficiency than their own (Daws, 2009). Moreover, there is increasing awareness of “uptake-2” transporters in monoamine clearance (Gasser and Daws, 2017a,b). These are classified as transporters with low-affinity, but high-capacity to take up monoamines, and include organic cation transporters (OCTs) and the plasma membrane monoamine transporter (PMAT).

Of the three OCT subtypes (OCT1, OCT2, OCT3), OCT2 and OCT3 are most widely expressed in brain (Amphoux et al., 2006; Vialou et al., 2008; Gasser et al., 2009; Couroussé and Gautron, 2015). PMAT is also widely expressed in brain, including limbic regions (Engel et al., 2004; Dahlin et al., 2007). Each of these transporters is capable of clearing serotonin, norepinephrine, and dopamine from extracellular fluid, albeit with varying affinities (Koepsell et al., 2007; Duan and Wang, 2010; Wang, 2016). Emerging evidence indicates that, in adult mice, OCT3 may be a novel target for the development of new antidepressants. For example, the non-selective OCT/PMAT blocker decynium-22 produced robust antidepressant-like effects in adult SERT heterozygous and knockout mice, which have upregulated OCT3 expression, but was without effect in SERT wildtype mice (Baganz et al., 2008). Subsequently, Horton et al. (2013) showed that decynium-22 augmented the antidepressant-like effect of a sub-effective dose of the SSRI fluvoxamine, and that this effect was attenuated in OCT3 knockout mice. Recently, novel decynium-22 analogs were synthesized and characterized. Unlike decynium-22, which lacks activity at SERT, NET, and DAT, analogs with activity at OCT3, *and modest* activity at SERT, were found to produce stand-alone antidepressant-like effects in wildtype mice. Together with the findings of Horton et al. (2013), these results support the notion that dual SERT/OCT3 blockers may be more effective antidepressants than SSRIs alone (Krause-Heuer et al., 2017; Fraser-Spears et al., 2019). In addition, studies using OCT2 knockout mice provide some evidence that OCT2 may also be a new pharmacological target for mood disorders (Bacq et al., 2012).

These findings in adult mice raise the possibility that OCTs and/or PMAT may play a mechanistic role in poor response of children and adolescents to SSRIs relative to adults. For example, based on SERT expression studies described earlier, it appears that SERT expression and perhaps function is lower in juveniles and adolescents than in adults. Putatively, serotonin clearance might be driven more by OCTs/PMAT during these post-natal periods, or perhaps these “uptake-2” transporters could be transiently expressed at greater functional levels than in adults. Though no data currently exists regarding the ontogeny of OCTs and PMAT in brain, in the periphery, OCT1 mRNA expression reaches adult levels by P15 in kidney and by P22 in liver (Alnouti et al., 2006). While it is difficult to translate these findings to changes that may occur in brain, these data support the idea that in juveniles and adolescents, OCTs and/or PMAT may already be at adult levels, or perhaps transiently expressed at higher levels. In this scenario, OCTs/PMAT could conceivably buffer any increase in serotonin following an SSRI, thereby reducing, or preventing therapeutic benefit (Daws and Gould, 2011). Studies using adult SERT heterozygote mice, which afford a murine model of humans carrying a common low expressing variant of the SERT gene, lend support to this premise. OCT3 expression is increased in these mice, presumably to compensate for reduced SERT expression/function (Baganz et al., 2008). As in humans carrying this low expressing SERT gene variant (Kronenberg et al., 2007), SERT heterozygote mice are less responsive to the antidepressant-like effect of escitalopram than SERT wildtype mice (Mitchell et al., 2016). However, SERT heterozygote mice are responsive to the antidepressant-like effect of decynium-22 (OCT/PMAT blocker), whereas SERT wildtype mice are not.

Although a role for OCTs/PMAT in antidepressant-like response of juveniles and adolescents awaits empirical verification, evidence in hand supports further investigation of these transporters in regulating homeostasis of serotonin neurotransmission (as well as that of other monoamines), during juvenile and adolescent periods.

## Conclusion

This review of current literature examining clinical and preclinical antidepressant efficacy of SSRIs in juveniles and adolescents reveals many knowledge gaps. These range from a need to better understand the ontogeny of neural systems known to be involved in antidepressant action in adults (in both humans and rodents), to a need for improved preclinical models in which to study antidepressant efficacy (across all ages). Although the utility of SSRIs in the treatment of pediatric depression is not clear from clinical studies, preclinical research (in spite of its current limitations) is providing valuable insights. Results from preclinical studies examining the acute and chronic antidepressant-like effects of SSRIs share remarkable similarities with clinical outcomes, supporting the use of paradigms such as the TST and FST as valuable tools to screen novel compounds for their potential therapeutic utility in these young populations. Studies of SERT expression and function, though few, are revealing a striking dichotomy in the effect of chronic SSRI treatment on SERT expression. In adults SERT is downregulated, whereas in juveniles and adolescents, SERT is upregulated. Moreover, there is growing evidence for a role of OCTs and PMAT in regulating monoamine neurotransmission. Literature reviewed in this focal area of “transporters for serotonin” encourages further research into what appears to be fundamental differences in SERT, and putatively other transporters capable of serotonin uptake, between juvenile and adolescents on the one hand, and adults on the other. In doing so, novel targets for the improved treatment of depression in children and adolescents should be revealed.

## Author Contributions

LD conceived the content. LD and MB wrote the manuscript.

## Conflict of Interest Statement

The authors declare that the research was conducted in the absence of any commercial or financial relationships that could be construed as a potential conflict of interest.
